# Role of Moesin in Renal Fibrosis

**DOI:** 10.1371/journal.pone.0112936

**Published:** 2014-11-18

**Authors:** Yong-Xi Chen, Wen Zhang, Wei-Ming Wang, Xia-Lian Yu, Yi-Mei Wang, Min-Jun Zhang, Nan Chen

**Affiliations:** 1 Department of nephrology, Ruijin Hospital, Shanghai Jiaotong University, school of medicine, Shanghai, PR China; 2 Animal Experiment and Research Center, Ruijin Hospital, Shanghai Jiaotong University, school of medicine, Shanghai, PR China; Biomedical Research Foundation of the Academy of Athens, Greece

## Abstract

**Background:**

Renal fibrosis is the final common pathway of chronic kidney disease (CKD). Moesin is a member of Ezrin/Radixin/Moesin (ERM) protein family but its role in renal fibrosis is not clear.

**Method:**

Human proximal tubular cells (HK-2) were stimulated with or without TGF-β1. Moesin and downstream target genes were examined by real-time PCR and western blot. Phosphorylation of moesin and related signaling pathway was investigated as well. Rat model of unilateral ureteral obstruction (UUO) was established and renal moesin was examined by immunohistochemistry. Moesin in HK-2 cells were knocked down by siRNA and change of downstream genes in transfected HK-2 cells was studied. All animal experiments were reviewed and approved by the Ethics Committee for animal care of Ruijin Hospital.

**Result:**

HK-2 cells stimulated with TGF-β1 showed up-regulated level of α-SMA and down-regulated level of E-Cadherin as well as elevated mRNA and protein level of moesin. In rat model of UUO, renal moesin expression increased in accordance with severity of tubulointerestital fibrosis in the kidneys with ureteral ligation while the contralateral kidneys were normal. Further study showed that TGF-β1 could induce phosphorylation of moesin which depended on Erk signaling pathway and Erk inhibitor PD98059 could block moesin phosphorylation. Effects of TGF-β1 on moesin phosphorylation was prior to its activation to total moesin. RNA silencing studies showed that knocking down of moesin could attenuate decrease of E-Cadherin induced by TGF-β1.

**Conclusion:**

We find that moesin might be involved in renal fibrosis and its effects could be related to interacting with E-Cadherin.

## Introduction

Chronic kidney disease (CKD) has received increased attention not only for its socioeconomic burden but also for its impact on the global public health. Irrespective of the underlying causes, progressive CKD often leads to renal fibrosis which is characterized as glomerulosclerosis and tubulointerstitial fibrosis histologically, and end-stage renal disease (ESRD) that requires costly renal replacement therapy clinically [1], [2]. Given this background, further investigating molecular details of renal fibrosis would help to provide comprehensive understanding of the disease and therapeutic strategies.

Moesin is a member of Ezrin/Radixin/Moesin (ERM) protein family that acts as cross linker between plasma membrane and the actin cytoskeleton [3], [4]. It belongs to a superfamily whose prototype is band 4.1 which share a common domain called FERM domain (Four point one Ezrin, Radixin Moesin) [3], [5]. Moesin and other ERM proteins are encoded by three genes in mammals that give rise to a single protein species and their expression is almost ubiquitous [6]–[8]. Structural analysis suggests that moesin as well as other ERM proteins regulate cell morphogenesis, adhesion, and migration by regulating actin cytoskeleton remodeling [6], [9]. To our knowledge, not many studies have been published on ERMs in kidney diseases and most of which focus on interaction between ERMs and advanced glycation end products (AGE) in pathogensis of diabetic kidney diseases [10], [11]. The role of ERMs in other renal diseases is still unclear.

In our previous proteomic study [12], we demonstrated that phosphorylation of moesin was involved in the transforming growth factor-β1 (TGF-β1) induced human tubular epithelial cell injury, but the molecular details of moesin were not fully investigated. Considering the important profibrotic effects of TGF-β1 in renal tubulointerestital fibrosis, we therefore study the role of moesin in such process so as to provide novel therapeutical targets for renal fibrosis and CKD.

## Methods

### Ethics Statement

All experiments involving rats were reviewed and approved by the Ethics Committee for animal care and use of research center for experimental medicine of Ruijin Hospital.

### Cell Culture

Human proximal tubular cells (HK-2, CRL-2190) obtained from ATCC were grown in keratinocyte serum-free media (KSFM, Invitrogen) supplemented with 0.05 mg/ml bovine pituitary extract (BPE, Invitrogen) and 5 ng/ml epidermal growth factor (EGF, Invitrogen) in a 37°C incubator with 5% CO_2_ as we previously described [12].

### RT-PCR and Real-Time RT-PCR

Total RNA was isolated from HK-2 cells or kidney tissues with TRIzol reagent (Invitrogen) following the manufacturer's protocol. Reverse transcription was performed according to the manufacturer's protocols with standard reagent (Promega). Real-time PCR amplification was performed using SYBR Green master mix (Toyobo, Japan) and Real-Time PCR (ABI). Primers were synthesized by invitrogen and listed in Table 1. Cycling conditions of Human α-SMA and E-Cadherin were initially denaturated at 95°C for 10 min, followed by 40 cycles consisting of a 15-second denaturation interval at 95°C and a 60-second interval for annealing and primer extension at 60°C as we previously reported [12]. Condition for human moesin includes denaturated at 48°C for 3 min and denaturated at 95°C for 10 min, followed by the same cycling conditions as human α-SMA. Condition for rat moesin includes denatured at 95°C for 3 min, followed by 45 cycles consisting of a 30-second denaturation interval at 95°C and a 30-second at 60°C, 30 second at 72°C and 10 min extension at 72°C. Condition for rat GAPDH includes denatured at 95°C for 30 seconds followed by 40 cycles consisting of 95°C for 15 seconds, and 60°C for 40 seconds. Condition for rat TGF-β1 and collagen type I includes 10 min at 95°C, followed by 40 cycles consisting of denatured at 95°C for 15 seconds, 20 seconds at 60°C for combined annealing, and 10 seconds at 72°C for extension. Relative amounts of mRNA were normalized by GAPDH and calculated using the delta-delta method from threshold cycle numbers.

**Table 1:** Real-time PCR primers used in the current study.

Gene	Primers(5′-3′)
Human moesin [36]	Forward: TGTAAACCAGAGAGCTGCTGG
	Reverse: GAAGAGCACACATGAGACAGAGAA
Human α-SMA [37]	Forward: GGGAATGGGACAAAAAGACA
	Reverse: CTTCAGGGGCAACACGAA
Human E-Cadherin [37]	Forward: ACCCCCTGTTGGTGTCTTT
	Reverse: TTCGGGCTTGTTGTCATTCT
Human GAPDH	Forward: CAGGGCTGCTTTTAACTCTGGTAA
	Reverse: GGGTGGAATCATATTGGAACATGT
Rat moesin [38]	Forward: CTGCGGGCTGATGCTATGG
	Reverse: GCAGGGTCTTGTATTTGTCTCGTC
Rat TGF-β1 [39]	Forward: CAACCCGGGTGCTTCCGCAT
	Reverse: TGCTCCACCTTGGGCTTGCG
Rat Collagen type I [39]	Forward: CACCTCCGGACGGAGCAGGA
	Reverse: CTCTTTGCGGCTGGGGTGGG
Rat GAPDH	Forward: TGAACGGGAAGCTCACTGG
	Reverse: TCCACCACCCTGTTGCTGTA

α-SMA: smooth muscle-alpha actin.

GAPDH: glyceraldehyde-3-phosphate dehydrogenase.

### Western Blot

HK-2 cells were lysed in sample buffer containing RIPA and phenylmethanesulfonyl fluoride (PMSF). Protein concentrations were determined by BCA protein assay kit (Pierce). Thirty micrograms of protein extract was separated by SDS-PAGE gel and transferred onto nitrocellulose membranes (Millipore). The blots were blocked with 5% nonfat dry milk in Tris-buffered saline containing 0.05% Tween 20 (TTBS) at room temperature for 1 hr, followed by incubation with antibodies against primary antibodies at 4°C overnight. After three washes, the membrane was then incubated with horseradish peroxidase (HRP)-conjugated secondary antibodies (Kirkegaard & Perry Laboratories) and visualized with ECL kits (GE Amersham). The primary antibodies that were used included anti-α-SMA antibody(1∶1,000, Sigma), anti-E-cadherin antibody (1∶500, BD Pharmigen), anti-moesin antibody (1∶1000, Cell Signaling), anti-Erk 1/2 antibody (1∶1000, Cell Signaling), anti-pErk 1/2 antibody (1∶1000, Cell Signaling), anti-pERM antibody (1∶1000, Cell Signaling) and anti-β-actin antibody (1∶10000, Sigma Aldrich).

### Inhibiting of moesin by siRNA

We designed three pairs of shRNA-moesin sequences and shRNAs were synthesized by Shanghai GenePharma (Shanghai, China). We then incorporated them respectively into a lentiviral vector and generated viral particles using viral packaging technology. PC12 cells were transfected with the viral particles before RNA extraction for determining knockout efficiency. Infected HK-2 cells with the highest knockout efficiency were chosen for subsequent experiments. In the preliminary study, we identified the shRNA with the most optimal inhibitory effect and used for current study. The sequence of the shRNA used in current study was: 5′ GCAAACTCAGCCTCAATAAGC 3′.

### Animal model

Unilateral ureteral obstruction (UUO) rat model was induced in male Sprague-Dawley (SD) rats (220 to 250 g; Animal center of Shanghai Institutes for Biological sciences, Shanghai, China) by ligation of the left ureter. Briefly, UUO rats (*n* = 15) were under isoflurane anesthesia, a midline abdominal incision was made and the left ureter was dissected out. The ureter was ligated at approximately 1 cm below the renal hilum with 3-0 silk suture. The abdominal wound was then closed. Control rats (*n* = 15) underwent abdominal incision and approximation with no ligation of the ureter. Rats were maintained in the animal facility with free access to water and standard food. At indicated time (0, 7, and 14 d after ureteral ligation), 5 rats from each groups were sacrificed under isoflurane anesthesia. Both left and right kidneys were used for further studies. The right kidney from the rats that had undergone left unilateral ligation was also used as another control.

### Semiquantitative assessment of tubulointerestital fibrosis

Rat kidney sections were stained with Masson trichrome and then assessed of collagen deposition in the light microscope. The fibrotic area was assessed by scoring semiquantitatively as viewed from a light microscope using x20 flat-field objective on the randomly selected four different regions on each section and then processed by the ImageJ software(National Institute of Health). All bar graphs represent Masson positively stained areas processed by ImageJ software.

### Immunohistochemistry

Rat kidneys were fixed with 4% paraformaldehyde and embedded in paraffin. Four-micrometer sections were cut and blocked with 10% goat serum/PBS for 30 min at room temperature. The sections were then incubated with anti moesin antibody (Cell Signaling) at 4°C overnight. Followed by being washed and incubated with biotinylatedanti-IgG secondary antibody (Dako) for 20 min. Biotin was identified by using streptavidin coupled to horseradish peroxidase and was visualized with diaminobenzidine (EnVision Detection Systems, Dako). Sections were viewed and imaged with a spot-cam digital camera (Carl Zeissy).

### Statistics

Statistical analysis was performed using SPSS 11.0 for windows (SPSS Inc., Chicago, IL, USA). Data were presented as mean ± SD, unless otherwise indicated. Differences among different groups were compared using one-way ANOVA. p value <0.05 was considered statistically significant.

## Results

### Moesin is elevated under TGF-β stimulation in human tubular epithelial cells

**Figure 1:**
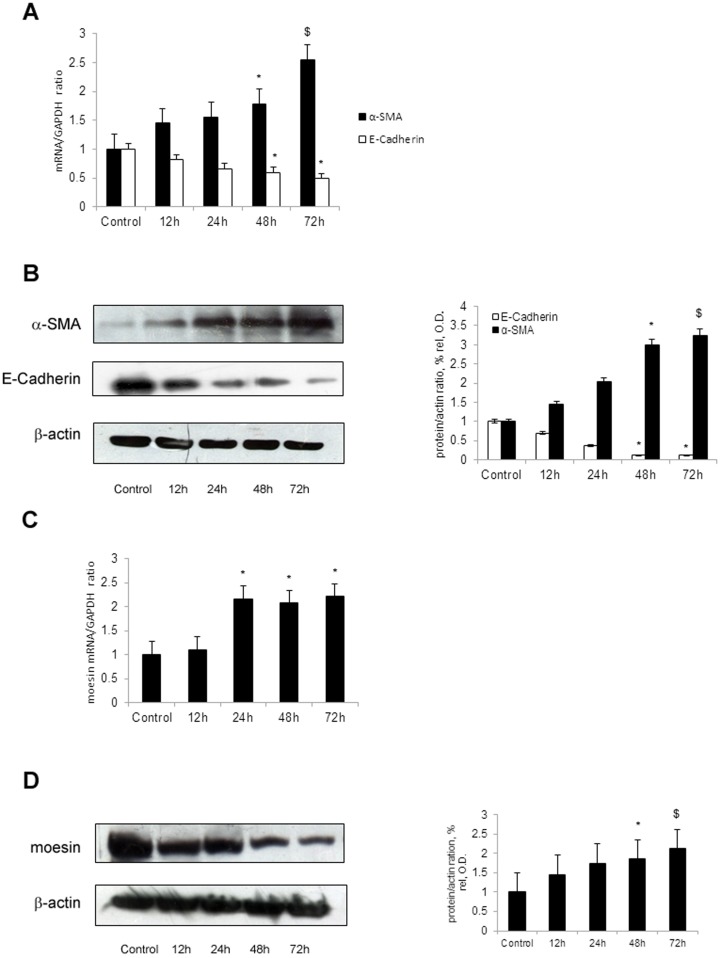
TGF-β1 up-regulates moesin and α-SMA, down-regulates E-Cadherin in HK-2 cells. HK-2 cells were maintained in the absence or presence of TGF-β1 (5 ng/ml) for various hours. The cells treated with TGF-β1 presented with up-regulated expression of α-SMA and down-regulated expression of E-Cadherin by real-time PCR (A) and western blot (B) in comparison with control. TGF-β1 also upregulated moesin expression in HK-2 cells for indicated time period. The expression of moesin was determined by real-time PCR (C) and western blot (D). β-actin was used to verify equivalent loading. Densitometrical analysis and real-time PCR results shown were results from three independent cell preparations. Western blot showed the results from one of three independent preparations. * p<0.05 versus Control; ^$^ p<0.01 versus Control.

As shown in **Figure 1**(A, B), HK-2 cells stimulated with TGF-β1 showed up-regulated level of cytoskeletal protein α-SMA and down-regulated level of mesenchymal adhesion protein E-Cadherin which were consisted with renal tubular epithelial cell injury. By using real-time PCR and western blot, we demonstrated that HK-2 cells stimulated with TGF-β1 presented with elevated mRNA and protein level of moesin (**Figure 1**C, D). These results indicated that induction of moesin by TGF-β1 was accompanied by the tubular epithelial cell injury.

### Moesin expression is elevated in the ligated kidneys of UUO rats

**Figure 2:**
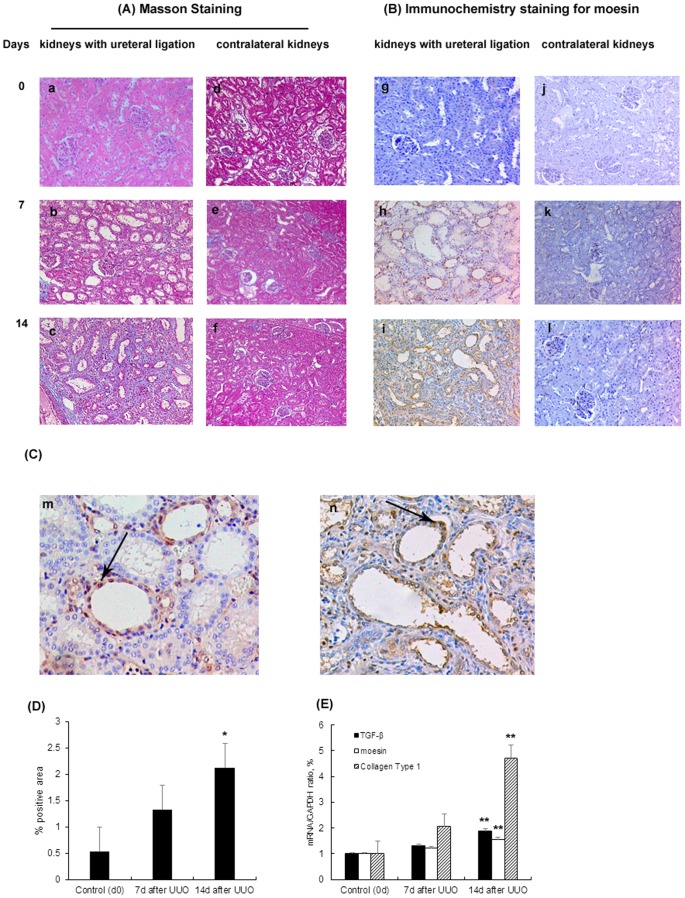
Expression of moesin in rat model of UUO. Kidney histology showed the histological injury of the rats. The rats had left kidneys ureteral ligation and right kidneys were set as control. (A) On masson stained sections (x100), the kidneys were normal at day 0 (a). One week after the left ureteral ligation, rats developed minor tubulointerestital injury which included minor tubular atrophy and mild collagen deposition (b). Two weeks after the ligation, severe tubulointerestital injury was found, including severe tubular atrophy and large amount of collagen deposition (c). (B) Immunohistochemical staining (x100) in rats' kidneys showed that there was barely moesin detected in the kidneys at day 0. There was increase of moesin staining in the tubulointerestitium at day 7 and day 14 which were in accordance with tubulointerestital injury. (C) Immunohistochemical staining (x200) also showed that the expressions of moesin in the kidneys with ureteral ligation were mainly localized in renal tubular epithelia cells. Arrows indicated moesin positively stained tubular epithelia cells at day 7(m) and day 14(n) after UUO. (D) Quantification of tubulointerestital fibrosis by using ImageJ software for Masson positively stained areas. (E) TGF-β, collagen type I and moesin mRNA expression in rat kidneys with ureteral ligation by real time PCR. * p<0.05 versus control group (day 0); ** p<0.01 versus control group (day 0).

To further investigate role of moesin *in vivo*, we continued our study in rat model of UUO which was a well characterized animal model of renal fibrosis [13]. Our results (**Figure 2**) showed that kidney histology was normal in both kidneys in UUO rats at day 0. Seven days after the surgery, rats developed tubulointerstitial damage including tubular atrophy and interstitial fibrosis in the kidneys with ureteral ligation while the contralateral kidneys were normal. The tubulointerestital fibrosis was confirmed by increased TGF-β and Collagen type I mRNA level in rat kidneys. The immunohistochemistry staining demonstrated that renal moesin expression was barely detected at day 0 and remarkably upregulated after 7 days of surgery. The moesin expression increased in accordance with the severity of tubulointerestital fibrosis.

### Phosphorylation of moesin is induced by TGF-β and depends on Erk 1/2

**Figure 3:**
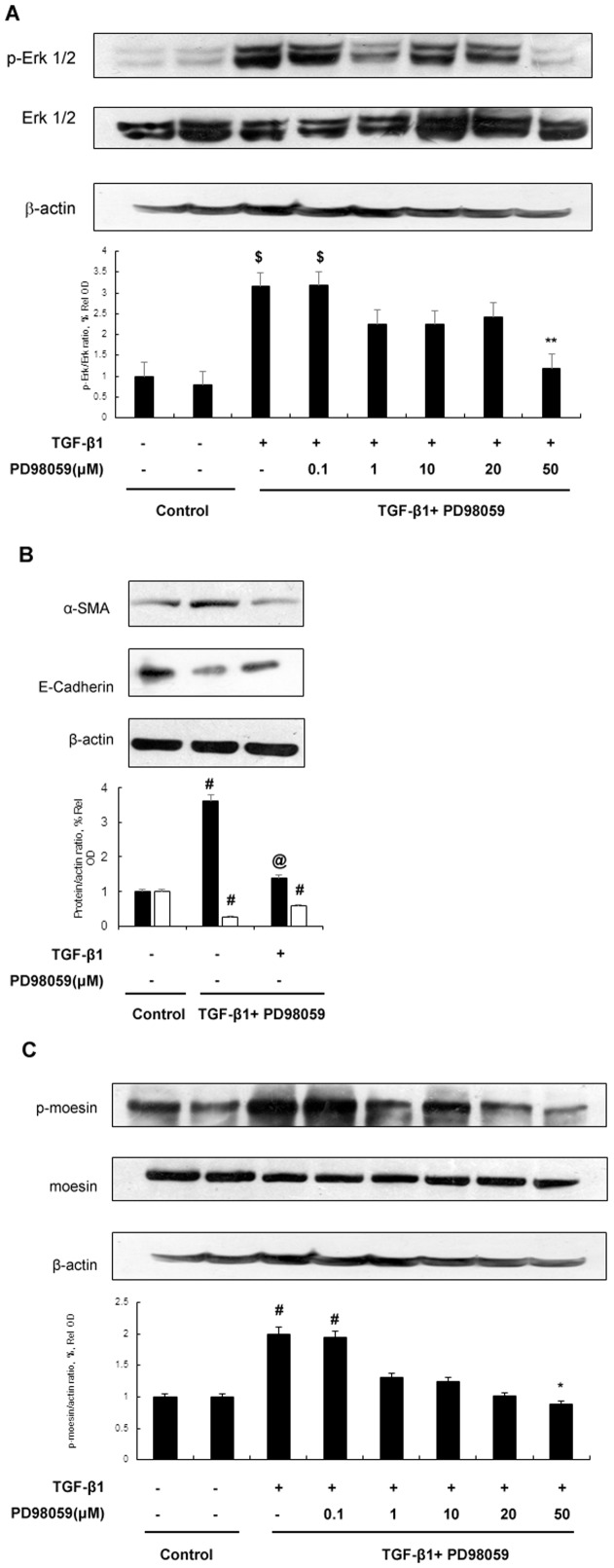
Erk activation is involved in TGF-β1 induced phosphorylation of moesin. (A) HK-2 cells that were treated with or without TGF-β1 (5 ng/ml) as well as Erk phosphorylation inhibitor PD98059 for 2 hrs. Immunoblots of lysates from HK-2 cells showed that TGF-β1 could induce phosphorylation of Erk 1/2. PD98059 could block Erk phosphorylation. Western blots results represented one of three independent cell preparations and results of densitometrical anayalsis. $ p<0.01 *versus* control group; ** p<0.05 *versus* TGF-β(+)/PD98059(−) group;(B) HK-2 cells that were treated with or without TGF-β1 (5 ng/ml) as well as PD98059 (50 µM) for 48 hrs. Blocking Erk phosphorylation could counteract effects of TGF-β1 on E-Cadherin and α-SMA in HK-2 cells. # p<0.05 *versus* control group; @ p<0.05 versus TGF-β(+)/PD98059(−) group. (C) HK-2 cells that were treated with or without TGF-β1 (5 ng/ml) as well as Erk phosphorylation inhibitor PD98059 for 2 hrs. The cells treated with TGF-β1 could induce phosphorylation of moesin without affecting moesin protein expression. Blocking Erk phosphorylation by PD98059 could inhibit phosphorylation of moesin in HK-2 cells.# p<0.05 *versus* control group; * p<0.05 *versus* TGF-β (+)/PD98059(−) group.

As it was shown that TGF-β1 could induce moesin *in vivo* and *in vitro*, and phosphorylation of moesin could also be induced by TGF-β1 [12], we next investigated the possible signaling pathway involved in its phosphorylation. As shown in **Figure 3**, TGF-β1 could induce phosphorylation of moesin which depended on Erk signaling pathway. Erk inhibitor PD98059 could block moesin phosphorylation and counteract effects of TGF-β1 on α-SMA and E-Cadherin.

### Knockdown of moesin attenuates TGF-β induced downregulation of E-Cadherin

**Figure 4:**
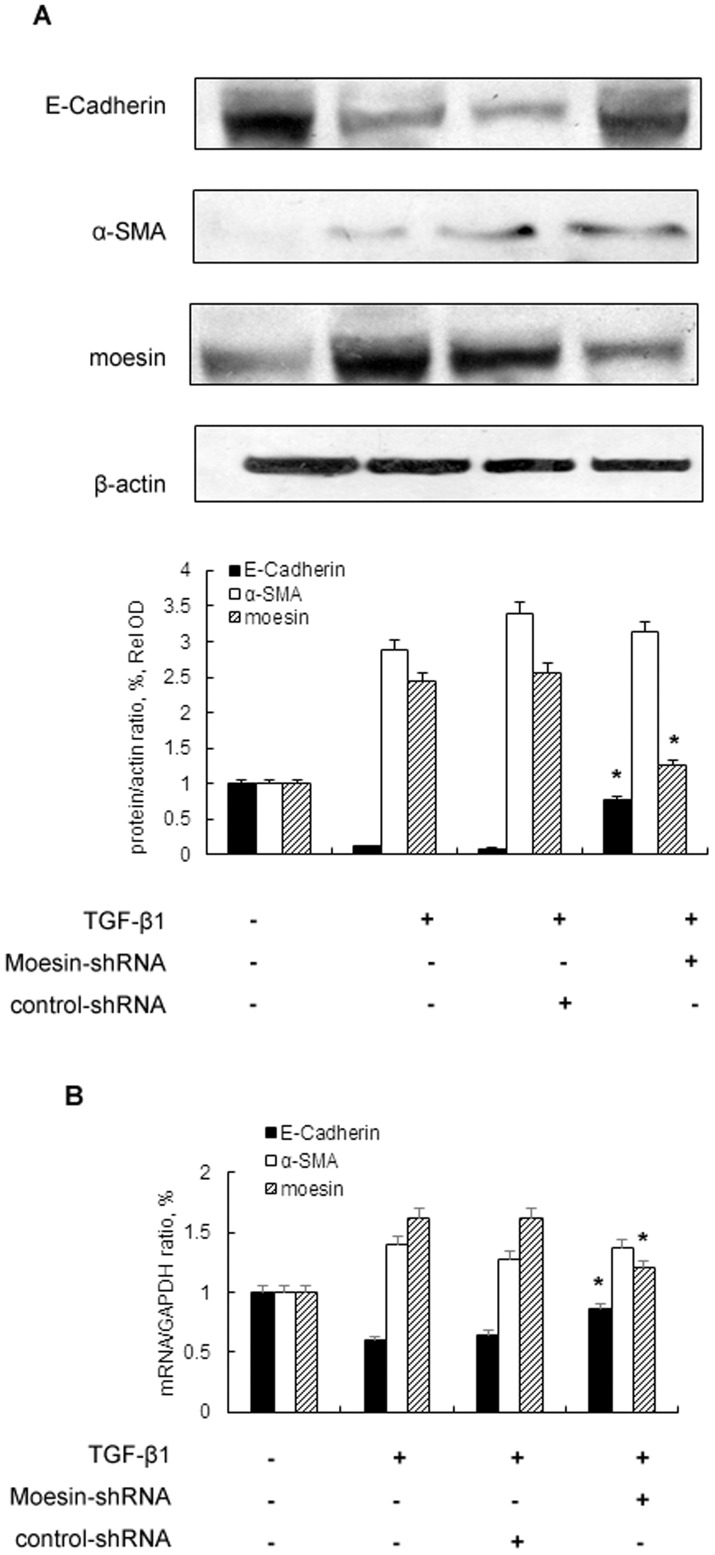
Knockdown of Moesin inhibits the expression of TGF-β induced downstream targets genes in HK-2 cells. HK-2 cells are transfected with control siRNA or moesin-siRNA followed by TGF-β1 (5 ng/ml) stimulation for 48 hrs. The expressions of α-SMA, E-Cadherin and moesin were determined by Western blot (A) and real-time PCR (B). Western blots analysis represented for one of three independent cell preprations.* p<0.05 *versus* TGF-β1 stimulated group and TGF-β1+control shRNA group.

In order to further investigate role of moesin in TGF-β1 stimulation, we constructed moesin shRNA to knockdown the expression of moesin. Our results (**Figure 4**) showed that knockdown of moesin could attenuate decreased expression of E-Cadherin induced by TGF-β1. However, the expression of α-SMA was not affected by suppression of moesin.

## Discussion

Despite of the initial causes, the progression of CKD would eventually lead to ESRD that is characterized as wide-spread renal fibrosis. The pathogenesis of renal fibrosis is a process of excessive accumulation and deposition of extracellular matrix components [14]. Several cellular events have been identified to be involved in such process including epithelial to mesenchymal transition (EMT), inflammatory cell infiltration and cell apoptosis [1], [14].

EMT has been firstly described in carcinogenesis in which the dedifferentiation of cells lose epithelial and get mesenchymal features [15]. In the study of renal fibrosis, tubular EMT during tissue injury could be associated with kidney fibrosis [16]. Tubular EMT is described as a process in which renal tubular cells lose their epithelial phenotype and acquire new characteristic features of mesenchyme [17]. By *in vitro* and *in vivo* studies, Liu and Yang [18] demonstrated that four key steps were involved in EMT which included: a) loss of epithelial cell adhesion, b) *de novo* α-smooth muscle actin expression and actin reorganization, c) disruption of tubular basement membrane, and d) enhanced cell migration and invasion. Importantly, TGF-β1 was a key mediator during these steps. In our previous and current study, we provided the evidence that TGF-β1 could induce α-smooth muscle actin expression and decrease adhesive protein expression which could contribute to EMT in human renal tubular epithelia cells [12]. Similar results were also found by O'Connor and colleagues which suggest cell adhesion and shape regulated EMT [19]. In the literature, EMT is known to contribute to the generation of renal fibrosis by inducing fibroblasts formation in injured tissues [16]. However recent studies have proposed different mechanisms regarding role of EMT in renal fibrosis which suggest EMT might not actively contribute to fibrotic development [20], [21]. The epithelial stress including endoplasmic reticulum (ER) stress, dysregulation of energy metabolism and many others could trigger profibrotic phenotype on the epithelial cell without activating EMT genes [22]. In the study by Kriz and colleagues [20], the authors proposed that during decomposition of tubules, the cells gradually lost their contacts with the basement membrane and therefore lost some of the junctional proteins which might not be related to mesenchymal phenotype. Similar results were also found by Bielesz and colleagues [23]. Koesters *et al* [24] showed in their study that autophagy and fibrosis was found to be induced by TGF-β1 in renal tubules but not EMT. However in the study by Neil and colleagues [25], TGF-β1-induced EMT can occur independently of its proapoptotic effects. Since the results from the literature were controversial, more investigations should focus on the molecular details in renal fibrosis and EMT [26].

In our study, we demonstrated that moesin, which is a member of ezrin/radixin/moesin (ERM) family, was involved in the process of renal fibrosis. It is reported that ERM proteins are highly conserved and high identity is observed in the FERM domain of different species [9]. Previous studies have found that the N-terminal domain of ERM proteins mediates cell-cell and cell-extracellular matrix adhesion, while the C-terminal domain regulates cell membrane extensions and cell motility [4], [10]. Though ERMs share structural identity, few functional diversity has been observed [6]. Functional study has demonstrated that moesin is essential to maintain epithelial cells integrity [27] while ezrin is crucial to branching tubulogenesis in the epithelial cells [28]. Though the literature suggests moesin and other ERM proteins could promote epithelial plasticity for morphogenesis and migration, their role in renal fibrosis has not been fully investigated. In the current study, we demonstrated that TGF-β1 could induce moesin expression in human renal tubular epithelial cells and the expression of moesin in the kidney increased in a rat model of renal fibrosis. Further study demonstrated that inhibiting moesin expression could attenuate TGF-β1 induced loss of cell adhesion in human tubular epithelial cells but no altered expression of α-SMA was observed after silencing moesin. Although it has been found that moesin could regulate cytoskeleton remodeling [15], [19], our result is not contrary to established findings. One possible explanation is that moesin could regulate relocalization of α-SMA during TGF-β1 stimulation and therefore total level of α-SMA might not change [29]. Our results thus suggested moesin might be involved in renal fibrosis by interacting with adhesive molecules in renal tubular cells.

Apart from the structural feature of moesin that is related to cytoskeleton regulation, post-transcriptional modification is also essential to its activation and function. Several studies have demonstrated that phosphorylation of threonine 558 is necessary for moesin activation [30], [31]. Further investigations show that phosphorylation of moesin promotes F-actin binding and is crucial to the formation of certain cellular structures [32], [33]. In our previous study, we found that phosphorylation of moesin could be induced by TGF-β1 [12]. In current study, as shown in **Figure 3**, TGF-β1 could induce phosphorylation of moesin as early as 2 hours after stimulation but it didn't affect its total level at indicated time. Since moesin level was upregulated at 12 hours after TGF-β1 stimulation and its expression increased significantly thereafter (**Figure 1**), our results thus suggested that TGF-β1 could induce phosphorylation of meosin prior to its effects on total moesin. Taken together, we hypothesized that phosphorylation of moesin could be the initiator to moesin activation and such effect depended on Erk signaling pathway. Meanwhile our results also showed that inhibiting Erk pathway by PD98059 could suppress moesin phosphorylation. In the literature, ROCK or PKC inhibitor could also block moesin phosphorylation [34], [35]. However, we did not find those pathways involved in our study (data not shown). Since various designing protocols were applied in different studies, the molecular details of ERM phosphorylation could be different accordingly. Nevertheless, all the studies demonstrate that phosphorylation is essential to moesin activation.

In conclusion, our results showed that moesin might be involved during renal fibrosis. In view of the essential role of TGF-β in renal fibrosis and CKD, our findings offered novel insights into the mechanism underlying activation of TGF-β signaling and development of renal fibrosis. Our results suggested that moesin might be a novel target for attenuating renal fibrosis and could be served as possible strategy for treatment of CKD.
